# Plasma levels of D-dimer and fibrin degradation products correlate with bullous pemphigoid severity: a cross-sectional study

**DOI:** 10.1038/s41598-021-97202-w

**Published:** 2021-09-07

**Authors:** Sijia Wang, Mei Lu, Zijun Zhao, Xueting Peng, Liang Li, Chuantao Cheng, Min Fang, Yumin Xia, Yale Liu

**Affiliations:** 1 Department of Dermatology, The Second Affiliated Hospital, Xi’an Jiaotong University, 157 Xiwu Road, Xincheng District, Xi’an, 710004 Shaanxi Province China; 2 Vanderbilt University School of Medicine, Nashville, TN USA; 3 Department of Thoracic Surgery, The Second Affiliated Hospital, Xi’an Jiaotong University, Xi’an, China; 4 Department of Health Checkup, The Second Affiliated Hospital, School of Medicine, Xi’an Jiaotong University, Xi’an, China

**Keywords:** Biomarkers, Diseases, Medical research

## Abstract

Bullous pemphigoid (BP), the most frequent blistering dermatosis in the elderly, is associated with increased mortality. The severity of BP can be assessed by detecting the anti-BP180 immunoglobulin G (IgG) concentration, but the lab test is not available in many community clinics. BP patients are usually in a hypercoagulable state with increased levels of D-dimer and fibrin degradation products (FDPs). We aimed to evaluate the use of D-dimer and FDPs in assessing BP severity. We compared the levels of plasma D-dimer, plasma FDPs, eosinophil counts, eosinophil cationic protein, and serum anti-BP180 IgG concentration between 48 typical BP patients and 33 Herpes zoster (HZ) patients (control group). Correlational analyses were conducted to determine the relationships between the lab values and common BP severity markers. The plasma D-dimer and FDP levels were higher in BP patients than in HZ controls (D-dimer: 3297 ± 2517 µg/L vs. 569.70 ± 412.40 µg/L; FDP: 9.74 ± 5.88 mg/L vs. 2.02 ± 1.69 mg/L, respectively, *P* < 0.0001). Significant positive correlations were found between D-dimer/FDP levels and BP severity markers (i.e. anti-BP180 IgG concentration [D-dimer: *r* = 0.3928, *P* = 0.0058; FDP: *r* = 0.4379, *P* = 0.0019] and eosinophil counts [D-dimer: *r* = 0.3625, *P* = 0.0013; FDP: *r* = 0.2880, *P* = 0.0472]) in BP patients. We also found an association between FDP and urticaria/erythema lesions (*r* = 0.3016, *P* = 0.0372), but no other BPDAI components. In 19 BP patients with complete remission after systemic glucocorticoid treatment, D-dimer and FDP levels decreased post-therapy (D-dimer: 5559 ± 7492 µg/L vs. 1738 ± 1478 µg/L; *P* < 0.0001; FDP: 11.20 ± 5.88 mg/L vs. 5.13 ± 3.44 mg/L; *P* = 0.0003), whereas they did not in BP patients with treatment resistant. Plasma D-dimer and FDP are convenient markers to evaluate BP severity assistant on BPDAI and eosinophil counts. FDP is also helpful for inflammatory lesions in BP patients.

## Introduction

Bullous pemphigoid is a rare autoimmune subepidermal blistering disorder with an annual incidence estimation of 2.4–23 cases per million in the general population^1^. The bullae form due to autoantibodies against the structural components of hemidesmosomes (including BP180) at the dermal–epidermal junction and eosinophil infiltration into the superficial dermis^2^. BP affects individuals worldwide, especially patients over 70 years of age. Timely assessment and treatment can decrease the risk of morbidity and mortality associated with BP. Dermatologists cannot accurately assess BP severity based on skin examination alone yet they need to know the severity to prescribe effective treatment^3,4^. BP severity can be evaluated via anti-BP180 immunoglobulin G (IgG) concentration^5^, BPDAI^6^, and some newly reported markers, like eosinophil counts^7^ and eosinophil cationic protein (ECP) ^8^. However, given BP’s rarity, many community clinics in China do not have access to anti-BP180 immunoglobulin G (IgG) and ECP tests. There remains an urgent need to identify readily accessible tests that can assess BP severity combining with objective BPDAI and blood eosinophil counts.

D-dimer is a type of FDP whose levels can reflect the degree of coagulation activation and fibrin formation^9,10^. Increased plasma D-dimer and FDPs have been widely used to evaluate disease severity in a wide range of conditions, including COVID-19, Crohn’s disease, and pulmonary embolism^11–14^. Since BP patients are often in a hypercoagulable state with elevated levels of D-dimer and FDP^15^, we investigated whether plasma D-dimer and FDP levels of BP patients before treatment could be used to assess BP severity.

## Methods

### Patients

We assessed plasma D-dimer and FDP in 48 typical BP patients and 33 age- and gender-matched HZ patients. We used HZ patients as the control group to exclude advanced age as a confounder since plasma D-dimer and FDP levels tend to rise with age. The cross-sectional study was conducted at the Second Affiliated Hospital of Xi’an Jiaotong University (Shaanxi, China) after obtaining approval from its ethics committee. Typical BP was diagnosed based on clinical, histopathological, serological, and immunofluorescent features (Fig. S1). The study involved BP patients older than 18 years of age and without systemic glucocorticoid treatment before admission (Table 1). We excluded BP patients with vascular diseases and abnormal renal or liver function, which are known to affect the plasma D-dimer and FDP levels.

**Table 1:** Inclusion and exclusion criteria for a cross-sectional study of BP patients.

Inclusion criteria	Exclusion criteria
Age > 18 years old Availability of recent coagulation test results Clinically significant subepidermal blisters ≥ 5 mm in diameter (defined as cutaneous blisters or ruptured blisters with a flexible roof covering a moist base) Dermal-epidermal separation surrounded by eosinophils on pathology Anti-BP180 ≥ 9 U/mL Immunofluorescence showed IgG and/ or C3 deposit No systemic glucocorticoid treatment before admission	A history of Vascular disease (e.g., deep venous thrombosis and aortic dissection) Anticoagulation therapy Abnormal liver and kidney function Cardiovascular disease Diabetes mellitus Cerebrovascular disease Acute or chronic inflammatory disease Malignancy with or without treatment

Twenty-eight patients post systemic glucocorticoid treatment (methylprednisolone 40–80 mg/day at progressively tapered doses) were followed up. The corticosteroid treatment led to complete clinical remission (defined as the absence of new BP lesions for a minimum of four weeks with complete healing of the prior lesions), partial remission (defined as incomplete healing of the prior lesions), or non-remission (absence of healing and growth of ≥ 10 new BP lesions per day). The patients were given low-dose corticosteroids (≤ 30 mg/day) when re-evaluating the levels of D-dimer and FDP (seven to ten days after the beginning of corticosteroid treatment).

All patients gave informed consent. The trial registration number was ChiCTR1800017560. All methods were carried out in accordance with relevant guidelines and regulations.

### Biochemical detection

D-dimer and FDP levels were analyzed according to the manufacturer’s instruction with some important pre-analytical procedures^16^. In brief, we used straight needle venipunctures without a tourniquet and collected 3 ml blood to plastic BD tubes with a 3.2% buffered sodium citrate anticoagulant. The blood was mixed immediately after collection and then vertically delivered to the laboratory within 1 h by hand. Samples are centrifuged at room temperature at 1500×g for at least 10 min at room temperature to plasma. Then FDP level and D-dimer level were measured using a turbidimetric method using Sysmex kit on Siemens Sysmex CA-7000 System analyzers.

The eosinophil counts were obtained from the whole blood and measured by Laboratory Departments in the Second Affiliated Hospital of Xi’an Jiaotong University. Serum was obtained from blood and anti-BP180 IgG concentration(A MESACUP BP180-ELISA kit, MBL, Nagoya, Japan) and eosinophil cationic protein (ECP, Elabscience, Wuhan, China) levels in some patients were detected according to the manufacturer’s instruction. The concentration ≥ 9 U/mL is set as a cutoff value for BP180 IgG concentration, while the ECP levels in BP serum were calculated based on the standard curve.

### Severity assessment

The BPDAI was used to estimate the severity, including three parameters that constitute the total BPDAI activity (erosions/blisters, urticaria/erythema, and mucosal blisters/erosions) and total BPDAI damage (pigmentation)^17^. The BPDAI scores range from 0–360 for total BPDAI activity (maximum score of 120 for each activity parameter) and 0–12 score for damage, with higher scores indicating greater disease activity or damage. We separated the BPDAI activity components to analyze the correlation between the lesion types and the coagulation markers.

### Statistical analysis

We used GraphPad Prism version 8.0.1 software (GraphPad Software, La Jolla, CA) for all statistical analyses. Continuous data were expressed as mean ± SD. Groups were compared by the Fisher’s exact test, Chi-square test, or unpaired *t*-test. Correlations between two parameters were analyzed using Pearson’s correlation test. Differences were considered statistically significant at *P* < 0.05. “*”, “**”, and “***” represented *P* < 0.05, *P* < 0.01, and *P* < 0.001, respectively.

### Ethics approval and consent to participate

This clinical trial was approved by the Ethics Committee of the Second Affiliated Hospital, Xi’an Jiaotong University. All participants provided informed consent and signed the informed consent form.

### Consent for publication

Written informed consent for publication of the patients’ clinical details and/or clinical images was obtained from the patients/guardians of the patients.

## Results

### Descriptive analysis of the cohort

The higher frequency of hypercoagulable states in BP patients promotes us to explore the potential role of D-dimer and FDP in BP severity evaluation. In this study, a total of 48 BP patients (29 men [63.6%] and 19 women [36.4%]) and 33 HZ patients were enrolled. No statistically significant difference was found in sex or age between BP patients and HZ controls (*P* > 0.05, Table 2). And the average BP duration was 15.51 ± 45.73 months (range 0.2–304 months). More clinical information can be found in Table S1.

**Table 2:** Demographic characters of patients with HZ and BP.

Groups	HZ	BP	*P*-value
No	33	48	
Age	69.03 ± 8.87	71.17 ± 8.62	0.2820
**Gender**			0.4966
Male	17	29	
Female	16	19	
D-dimer (μg/L)	569.70 ± 412.40	3297 ± 2517	< 0.0001
FDP (mg/L)	2.02 ± 1.69	9.74 ± 5.88	< 0.0001
Eosinophil counts (× 10^9^/L)	0.11 ± 0.12	1.16 ± 1.83	0.0013

### Elevated D-dimer, FDP, eosinophil counts, and ECP in BP

**Figure 1:**
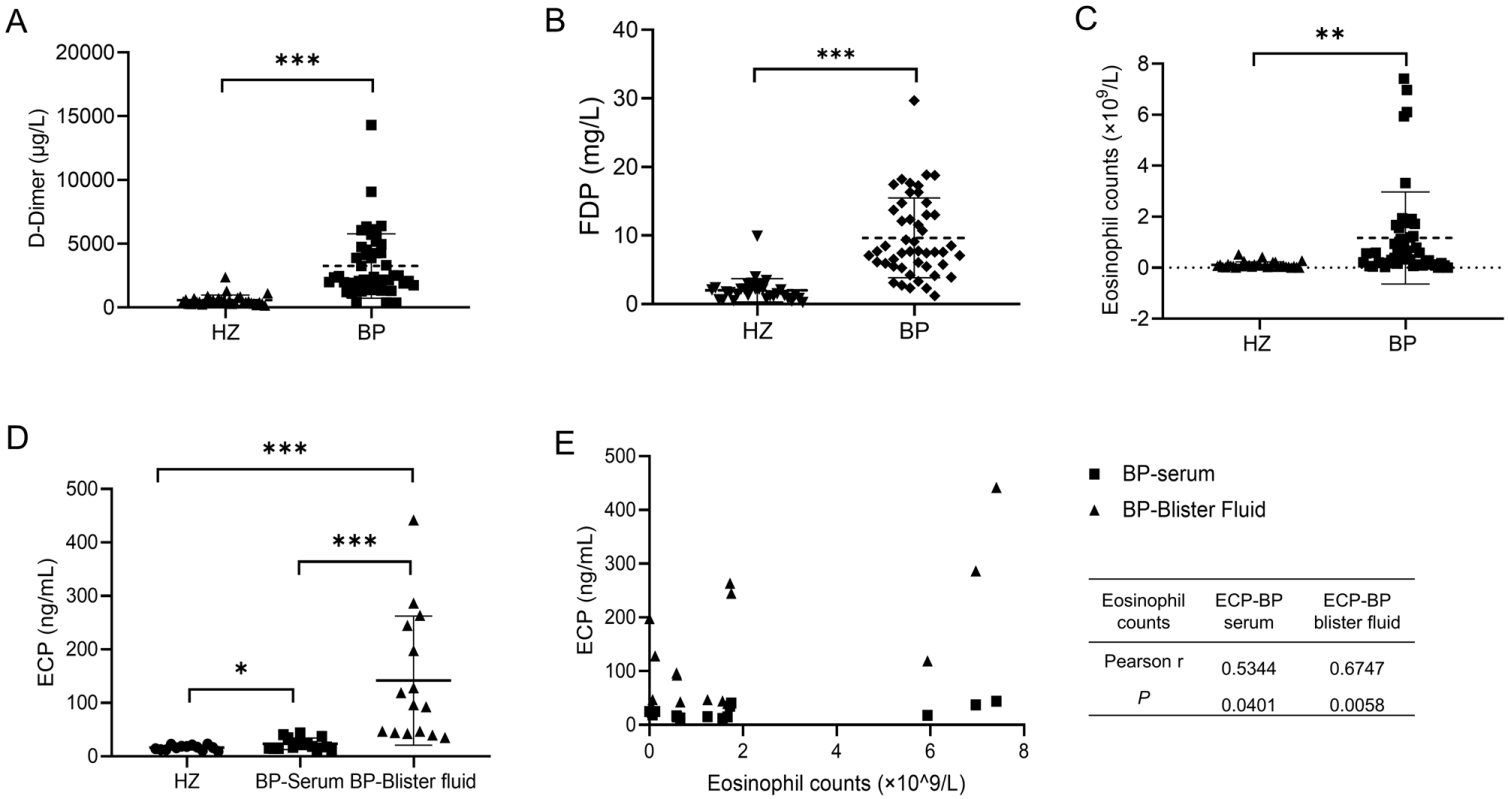
The levels of D-Dimer, FDP, and eosinophils in 48 BP patients and 33 HZ controls. (**A** and **B**) D-dimer and FDP levels were increased in BP patients compared to HZ controls; (**C**) Peripheral blood eosinophil counts in BP patients were higher than those in HZ controls; (**D**) Eosinophil cationic protein (ECP) expression was upregulated in BP serum and blister fluid than that in serum of HZ controls; (**E**) The correlation between eosinophil counts and serum/ blister fluid ECP levels. (*P < 0.05; **P < 0.01; ***P < 0.001). The solid lines represent upper and lower quantile while a dotted line in the middle symbolizes mean.

We first explored the levels of D-dimer and FDP levels in BP patients and found elevated plasma D-dimer (3297 ± 2517 µg/L vs. 569.70 ± 412.40 µg/L, *P* < 0.0001) and FDP levels (9.74 ± 5.88 mg/L vs. 2.02 ± 1.69 mg/L, *P* < 0.0001, Table 2 and Fig. 1A, B), compared to the age- and gender-matched HZ patients, validating the hypercoagulable states in BP patients.

As eosinophils are one of the key sources of tissue factor (TF)^18^ and participate in BP pathogenesis^19^, we also detected the eosinophil counts and found it is higher in BP patients than in HZ patients (1.16 ± 1.83 × 10^9^/L vs. 0.11 ± 0.12 × 10^9^/L, *P* = 0.0013, Table 2 and Fig. 1C), suggesting the role of eosinophils in hypercoagulable state of BP patients. Since activated eosinophils always release eosinophil granule proteins, especially ECP, we also detected the levels of ECP in serum and blister fluid of 15 BP patients. Our results revealed slightly increased ECP in BP serum (23.51 ± 10.74 ng/mL, *P* = 0.0356) and significantly increment in BP blister fluid (141.80 ± 120.70 ng/mL, *P* = 0.0009) than in HZ serum (16.42 ± 1.24 ng/mL, Fig. 1D), consistent with the results of Giusti et al.^8^ and Tedeschi et al.^20^. The positive correlation between eosinophil counts and serum (*r* = 0.5344, *P* = 0.0401) or blister fluid (*r* = 0.6747, *P* = 0.0058, Fig. 1E) ECP further validated the role of eosinophils in coagulation cascade of skin^20^.

### Anti-BP180 IgG concentration correlates with BPDAI

As anti-BP180 IgG and BPDAI are commonly used to evaluate the BP severity, we first validated these results in our samples and found increased anti-BP180 IgG concentration in BP patients (120.6 ± 66.0 vs 2.05 ± 1.89 U/mL, *P* < 0.001, Fig. S2A), with which total BPDAI score is positively correlated (*r* = 0.3251, *P* = 0.0242, Fig. S2B). To determine whether anti-BP180 IgG concentration is associated with certain lesion types, we analyzed the relationships between anti-BP180 IgG concentration and individual BPDAI components and discovered a moderate correlation with erosions/blisters (*r* = 0.3402, *P* = 0.0180), but not urticaria/erythema (*r* = 0.1261, *P* = 0.3931), pigmentation (*r* = 0.2797, *P* = 0.0542), and mucosal damage (*r* = -0.0833, *P* = 0.5734, Fig. S2C), which is consistent with van Beek N’s research^21^.

### D-Dimer, FDP, and eosinophil counts correlation with anti-BP180 IgG concentration and/or BPDAI

**Figure 2:**
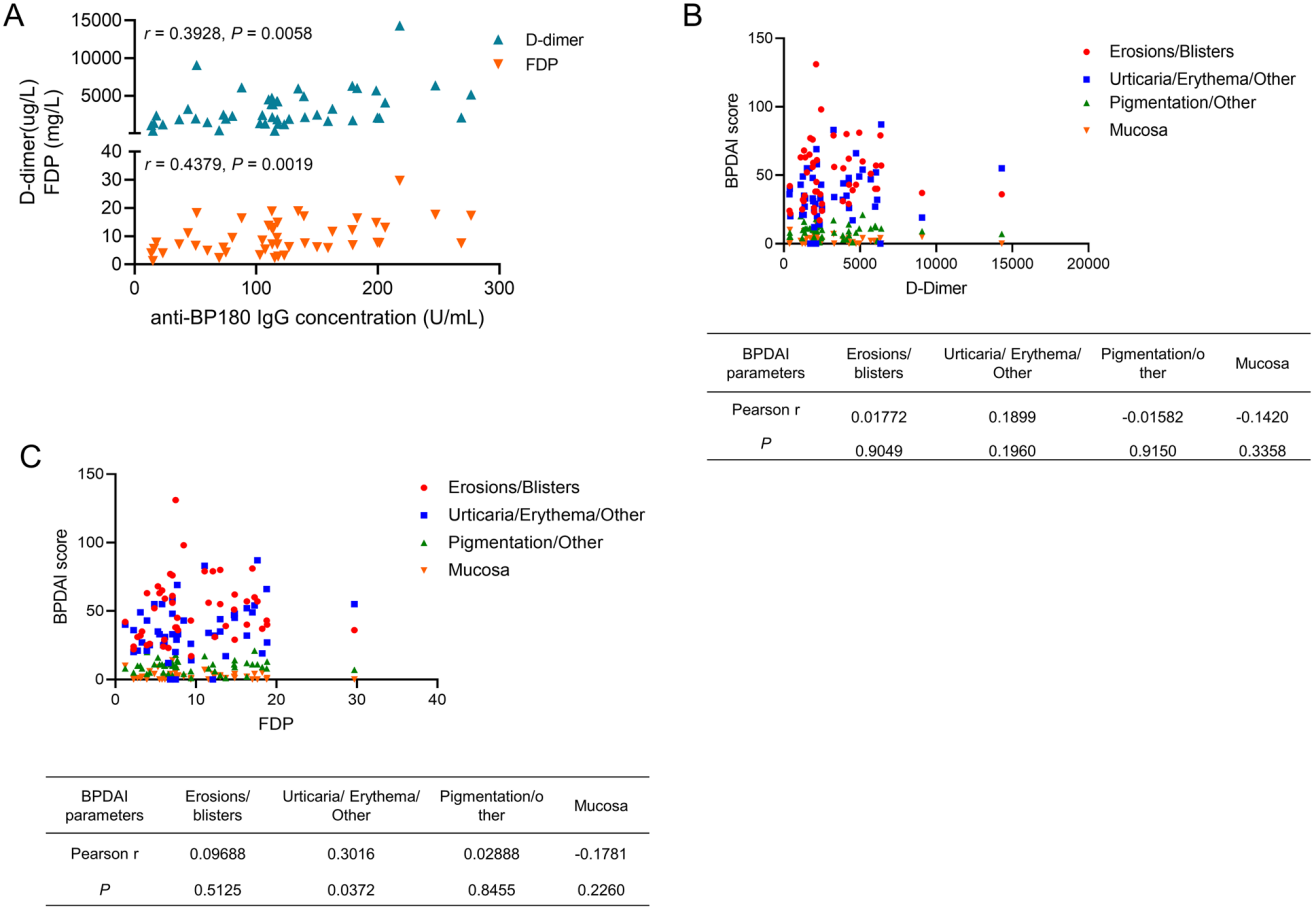
The correlation among D-Dimer, FDP, anti-BP180 IgGconcentrations, and BPDAI. (**A**) The anti-BP180 IgG concentration correlates with the levels of D-dimer and FDP; (**B** and **C**) The correlation between the levels of D-dimer and FDP and BPDAI.

Considering the critical roles of anti-BP180 IgG concentration and BPDAI, we further analyzed the correlation between the levels of anti-BP180 IgG concentration and BPDAI and levels of D-Dimer and FDP. Our results revealed serum anti-BP180 IgG concentration was positively correlated with the levels of plasma D-dimer (*r* = 0.3928, *P* = 0.0058) and FDP (*r* = 0.4379, *P* = 0.0019) (Fig. 2A). However, we did not identify correlations between the coagulation markers and individual BPDAI components except for a mild correlation between FDP and urticaria/erythema lesions (*r* = 0.3016, *P* = 0.0372, Fig. 2B, C).

**Figure 3:**
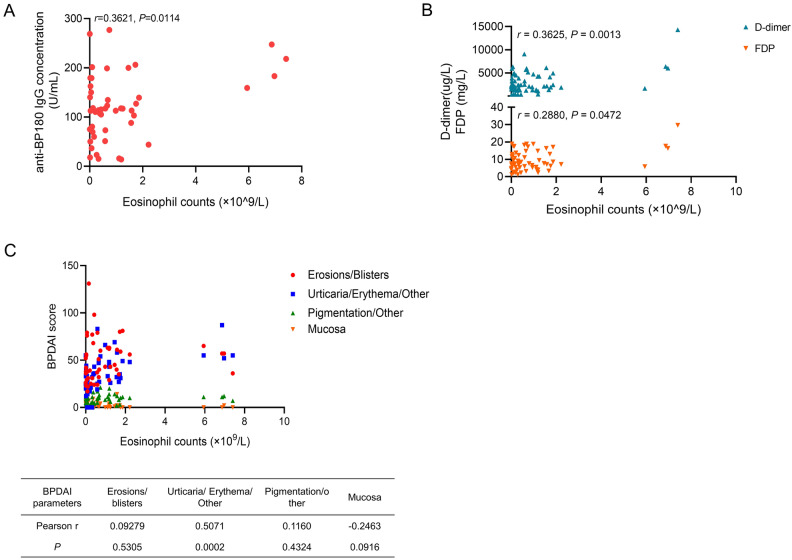
The correlation among eosinophil counts, anti-BP180 IgG concentrations, D-Dimer, FDP, and BPDAI. (**A**) The correlation between eosinophil counts and anti-BP180 IgG concentration; (**B**) The correlation of eosinophil counts and levels of D-dimer and FDP; (**C**) The correlation between eosinophil counts and BPDAI indexes.

Eosinophil counts are also evaluated and we detected a correlation between blood eosinophil counts and anti-BP180 IgG concentration (*r* = 0.3621, *P* = 0.0114, Fig. 3A), as well as plasma D-dimer (*r* = 0.3625, *P* = 0.0013) and FDP (*r* = 0.2880, *P* = 0.0472) levels (Fig. 3B). We also found that eosinophil counts were associated with urticaria/erythema lesions (*r* = 0.5071, *P* = 0.0002, Fig. 3C), but not other lesions.

Serum and blister fluid ECP in 15 patients were also analyzed to check the correlation with D-dimer and FDP. We found serum ECP (Fig. S3A) has no correlation with serum D-dimer (r = 0.3816, *P* = 0.1604) and FDP (r = 0.4475, *P* = 0.0944, Fig. S3A), but blister fluid ECP is correlation with them (D-dimer: r = 0.5307, *P* = 0.0418; FDP: r = 0.5920, *P* = 0.0201; Fig. S3B), suggesting eosinophil recruitment and ECP release in lesion area.

### Treatment response paralleled decreased D-dimer and FDP levels

**Figure 4:**
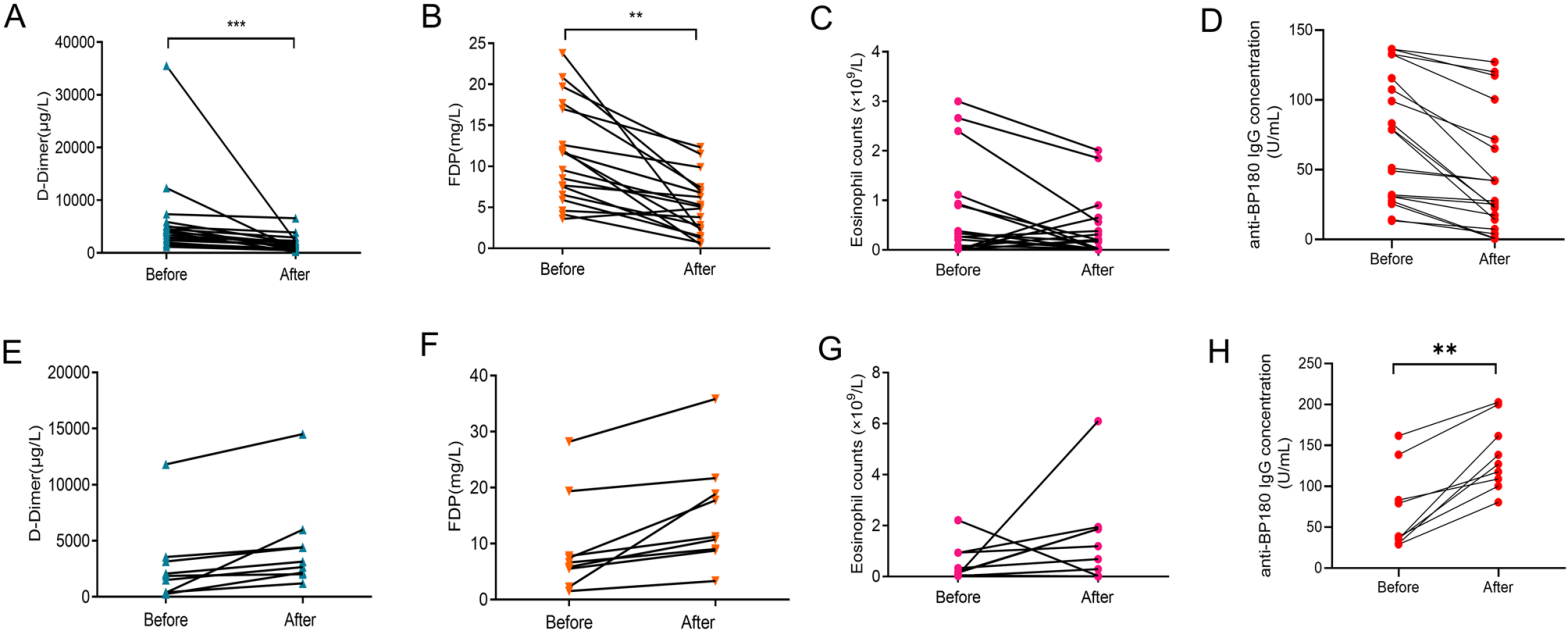
D-dimer, FDP, eosinophil counts, and anti-BP180 IgG concentrations in BP patients with treatment-effective and -resistant BP. Plasma D-dimer levels (**A**), FDP levels (**B**), eosinophil counts (**C**), and anti-BP180 IgG concentration (**D**) in 19 patients with treatment-effective BP were evaluated before and after immunosuppressive therapy. Marked reductions in these markers were observed after complete remission. Plasma D-dimer levels (**E**), FDP levels (**F**), eosinophil counts (**G**), and anti-BP180 IgG concentration (**H**) in nine patients with treatment-resistant BP were evaluated before and after immunosuppressive therapy. A slight elevation in these markers was observed without relief. (**P < 0.01; ***P < 0.001).

Since D-dimer and FDP are correlated with BP severity, we then investigate whether their levels will decrease after treatment. We, therefore, collected plasma D-dimer and FDP levels from 28 BP patients before and after therapy to check the changes. Among 28 patients, 19 patients with complete remission after therapy showed a marked reduction in plasma D-dimer levels (5559 ± 7492 µg/L vs. 1738 ± 1478 µg/L; *P* < 0.0001, Fig. 4A) and FDP levels (11.20 ± 5.88 mg/L vs. 5.13 ± 3.44 mg/L; *P* = 0.0003, Fig. 4B) as well as eosinophil counts (0.70 ± 0.98 mg/L vs. 0.42 ± 0.61 mg/L; *P* = 0.3028, Fig. 4C) and anti-BP180 IgG concentration (72.46 ± 45.14 mg/L vs. 45.92 ± 42.31 mg/L; *P* = 0.0697, Fig. 4D); nine patients with treatment-resistance had increased plasma D-dimer levels (2780 ± 3588 µg/L vs. 4503 ± 4032 µg/L; *P* = 0.3523, Fig. 4E), FDP levels (9.38 ± 8.71 mg/L vs. 15.22 ± 9.64 mg/L; *P* = 0.1968, Fig. 4F), eosinophil counts (0.49 ± 0.70 mg/L vs. 1.40 ± 1.83 mg/L; *P* = 0.1616, Fig. 4G), and anti-BP180 IgG concentration (70.27 ± 49.94 mg/L vs. 137.60 ± 42.83 mg/L; *P* = 0.0074, Fig. 4H), though some of the differences were not statistically significant, further suggesting the correlation between the levels of D-dimer and FDP and BP severity.

## Discussion

Identifying accessible tests that can assess BP severity in community clinics lacking anti-BP180 IgG concentration tests remains an urgent need. We report a positive association between two coagulation markers (plasma D-dimer and FDP) and known markers of BP severity including anti-BP180 IgG and eosinophil counts. The FDP is also associated with urticaria/erythema lesions, one component of BPDAI scores. These results suggest that the coagulation markers can be used to evaluate BP severity with BPDAI and eosinophil counts under the circumstances of no anti-BP180 IgG concentration test. FDP is also a helpful marker for inflammatory lesions in BP.

BP’s inflammatory lesions lead to activation of the coagulation cascade, resulting in elevated plasma D-dimer and FDP levels^22^. It is no surprise that D-dimer/FDP levels were increased in treatment-resistant patients but decreased in treatment responsive patients. Interestingly, we found that FDP levels are associated with urticaria/erythema in BP, which may occur for several reasons: (1) FDP can increase the vascular permeability and thereby induce wheals^23^; (2) FDP can initiate thrombin-dependent activation of mast cells^24^; (3) the urticaria/erythema associated with anti-BP180 IgE autoantibodies may increase the FDP level^25^; (4) FDP has a pro-inflammatory effect by increasing IL-6, TNF-α, and MCP-1^26,27^, which can also induce urticaria/erythema^28^.The failed association between D-dimer and BP lesions may suggest that the smaller fibrin degradation products do not contribute obviously to lesion formation^29^. The short half-life of D-dimer and the longer disease duration may lead D-dimer not closely connect to BPDAI^11^. Moreover, BP patients are enriched in serum anti-BP180 IgE, anti-BP230 IgG, and anti-BP230 IgE^9,10^, and D-dimer may correlate with them. Further investigations are necessary to validate these speculations.

Eosinophils are critical to BP pathogenesis. We have demonstrated increased eosinophil counts in BP, similar to other studies^7,8,30^, and found a correlation with BP severity. The positive correlation between eosinophil counts and D-dimer/FDP levels may be due to eosinophils’ role as major intravascular storage locations for tissue factor (TF)^18^, an initial factor of the extrinsic coagulation pathway. TF facilitates the early trans-endothelial migration of eosinophils, which can directly damage endothelial integrity. Eosinophils can also release ECP and alter the cutaneous microcirculation^31^, which validated the correlation of ECP and hypercoagulable states and explained the correlation we observed between eosinophils and urticaria/erythema lesions.

Autoantibodies to coagulation factors could modulate their activity^32^. Given that anti-BP180 autoantibody levels correlated with D-dimer/FDP levels, it may suggest that anti-BP180 IgG antibodies can activate coagulation factors. Anti-BP180 autoantibodies, especially IgG type, have been found to negate, alter, or promote the rapid clearance of clotting factors^32,33^. The anti-BP180 autoantibody may also mediate the formation of neutrophil extracellular traps (NETs) and induce a hypercoagulable state, previously seen in COVID-19 patients^34–36^. The precise mechanism of autoantibodies and hypercoagulation in BP requires further study.

Limitations of the study include a limited clinical recruitment site at an inpatient department of a single hospital, which may introduce selection bias. Additional medical centers and BP outpatients (usually expressing mild symptoms) should be considered in future studies. The selection criteria for the control group were not optimal, because blisters in HZ were usually limited and the magnitude of blisters can be mild compared to that of BP patients. The comparison of BP to HZ patients can potentially involve a risk of obtaining false positives in the statistical analyses. Larger sample size is needed to accurately assess the markers to confirm their clinical significance. In addition to anti-BP180 IgG, anti-BP180 IgE, anti-BP230 IgG, and anti-BP230 IgE are also associated with BP severity and their relationships with D-dimer/FDP levels should also be evaluated.

## Conclusions

In BP patients, plasma D-dimer and FDP levels are correlated with current BP severity markers including anti-BP180 IgG concentration, and eosinophil counts. Additionally, FDP is correlated to urticaria/erythema lesions. The two coagulation markers may be potential means to evaluate BP severity assistant on BPDAI and eosinophil counts. FDP could be more useful in the case of inflammatory BP.

## Supplementary Information

Supplementary Information.

## Data Availability

The datasets used and/or analyzed during the current study are available from the corresponding author on reasonable request.

## Abbreviations

BP: Bullous pemphigoid
BPDAI: Bullous pemphigoid disease area index
ECP: Eosinophil cationic protein
FDP: Fibrin degradation products
HZ: Herpes Zoster
IgG: Immunoglobulin G
