# Supplementary material for: Plasma levels of D-dimer and fibrin degradation products correlate with bullous pemphigoid severity: a cross-sectional study
Source: Sci Rep. 2021 Sep 7;11:17746. doi: 10.1038/s41598-021-97202-w (PMC8423823; doi:10.1038/s41598-021-97202-w)
Supplement: Supplementary file 1 — Supplementary Information. [file 41598_2021_97202_MOESM1_ESM.pdf]

Title: Plasma levels of D-dimer and fibrin degradation products correlate with bullous pemphigoid severity: a cross-sectional study

Keywords: D-dimer, fibrinogen/fibrin degradation products, bullous pemphigoid, severity

Authors: Sijia Wang[1]*, Mei Lu[1]*, Zijun Zhao[2], Xueting Peng[1], Liang Li[3], Chuantao Cheng[1], Min Fang[4], Yumin Xia[1#], Yale Liu[#1].

1. Department of Dermatology, The Second Affiliated Hospital, School of Medicine, Xi'an Jiaotong University, Xi'an, China

2. Vanderbilt University School of Medicine, Nashville, TN, USA

3. Department of Thoracic Surgery, The Second Affiliated Hospital, Xi'an Jiaotong University, Xi'an, China

4. Department of Health Checkup, The Second Affiliated Hospital, School of Medicine, Xi'an Jiaotong University, Xi'an, China

*These two authors contribute equally

#Corresponding author:

Yale Liu, M.D., Ph.D., The Second Affiliated Hospital, School of Medicine, Xi'an Jiaotong University, 157 Xiwu Road, Xincheng District, Xi'an, Shaanxi Province 710004, PR China. Tel.:86-29-8767-9327; Fax:86-29-8740-9503; Email: liuyale0703@xjtu.edu.cn.

Yumin Xia, M.D., Ph.D., The Second Affiliated Hospital, School of Medicine, Xi'an Jiaotong University, 157 Xiwu Road, Xincheng District, Xi'an, Shaanxi Province 710004, PR China. Tel.:86-29-8767-9327; Fax:86-29-8740-9503; Email: xiayumin1202@163.com.

Supplementary legends

Table S1 Clinical information of BP and HZ patients.

Fig. S1 Criteria for diagnosing BP. **A.** A typical clinical presentation of a patient with tense blisters on the skin; **B.** Histological section of subepidermal blisters with scattered eosinophil infiltration. IgG (**C**) and C3 (**D**) deposits along the basement membrane.

Fig. S2 Increased anti-BP180 IgG concentration in BP patients is correlated with BPDAI score. Anti-BP180 IgG concentration is in BP patients and HZ controls (**A**). The relation between anti-BP180 IgG concentration and total BPDAI score (**B**) or BPDAI components (**C**). (***$P < 0.001$)

Fig. S3 The relation between serum (**A**) or blister fluid ECP (**B**) and D-dimer or FDP levels.

Table S1 Clinical information of HZ and BP patients

HZ ID	Age (y)	Gender	Eosinophils (×10^9/L)	D-D (ug/L)	FDP (mg/L)	anti-BP180-IgG (U/mL)
HZ1	62	M	0.22	300	1.27	2.53
HZ2	89	F	0	890	3.99	1.86
HZ3	61	M	0.28	150	0.6	1.18
HZ4	68	M	0.26	550	2.18	1.18
HZ5	66	F	0.07	560	2.39	1.07
HZ6	76	F	0.08	480	1.39	0.51
HZ7	83	M	0.02	860	3.05	2.76
HZ8	70	M	0.12	320	0.5	2.98
HZ9	69	F	0.01	440	2.18	1.63
HZ10	61	F	0.03	190	0.24	1.41
HZ11	63	F	0.04	370	0.85	1.75
HZ12	78	F	0.14	410	1.15	0.84
HZ13	84	M	0.23	750	2.18	7.38
HZ14	58	F	0.42	280	0.46	6.87
HZ15	72	F	0.1	300	1.45	2.87
HZ16	65	M	0.52	290	1.2	0.51
HZ17	69	F	0.03	350	0.64	0.96
HZ18	66	M	0.09	1110	3.46	7.38
HZ19	59	M	0.09	590	2.08	4.11
HZ20	68	M	0.09	430	1.79	2.87
HZ21	73	M	0.09	2370	9.91	0.98
HZ22	86	M	0.04	730	2.43	0.93
HZ23	62	M	0.1	250	2.77	0.94
HZ24	60	M	0	500	1.27	0.62
HZ25	63	F	0.07	440	1.52	1.59
HZ26	83	M	0.05	730	2.87	1.36
HZ27	61	F	0.06	380	0.79	1.17
HZ28	67	F	0.05	630	2.21	0.40
HZ29	62	F	0.05	290	0.85	0.39
HZ30	60	M	0.03	600	2.08	0.60
HZ31	70	F	0.13	570	1.69	1.55
HZ32	62	F	0.08	400	2.18	1.33
HZ33	82	M	0.06	1290	2.9	3.14

BP ID	Age (y)	Gender	Course of disease	Eosinophil counts (×10^9/L)	D-D (ug/L)	FDP (mg/L)	anti-BP180-IgG (U/mL)	BPDAI				Total BPDAI
								Erosions/ blisters	Urticaria/ Erythema/ Other	Pigmentation/ other	Mucosa	
BP1	73	M	1m	0.09	2350	9.42	80.40	17	14	1	1	33
BP2	57	M	1m	0.02	1990	6.56	50.18	23	12	4	0	39
BP3	79	F	1y	0.11	430	2.26	69.53	22	20	5	1	48
BP4	69	M	3m	0.26	1210	3.91	23.18	25	21	4	0	50
BP5	75	F	15d	0.21	4520	13.71	110.33	39	17	1	1	58
BP6	84	M	3m	0.43	370	2.26	115.33	24	36	5	0	65
BP7	69	F	40d	0.33	3870	12.35	113.80	31	32	6	3	72
BP8	75	M	6m	0.01	2170	7.45	140.09	38	20	9	5	72
BP9	82	M	20d	0.09	2070	7.58	201.46	38	29	9	2	78
BP10	67	F	3m	0.68	5990	18.84	134.67	40	27	13	1	81
BP11	62	F	2m	1.18	4260	14.82	117.57	29	45	9	1	84
BP12	63	F	3m	0.01	2390	7.76	17.91	36	33	13	3	85
BP13	87	M	2y	0.33	1740	6.8	179.39	77	0	11	4	92
BP14	73	M	3y	0.03	3300	11.56	162.67	56	34	8	0	98
BP15	84	M	15d	0.02	390	1.21	15.03	42	40	8	10	100
BP16	57	F	5m	0.03	3900	13.01	112.56	55	44	3	3	105
BP17	83	M	3y	1.11	1400	5.48	15.88	63	33	11	0	107
BP18	56	M	7d	0.65	5710	14.75	198.82	51	47	11	2	111
BP19	61	F	6y	2.22	1890	7.08	117.41	56	48	10	0	114
BP20	63	M	5m	0.07	1890	7.08	36.71	76	33	3	2	114
BP21	70	M	5m	0.98	4740	18.79	112.89	43	66	8	0	117
BP22	63	F	2y	0	1520	4.84	59.97	52	55	11	4	122
BP23	65	M	5y	0.38	1330	5.28	107.38	68	35	16	9	128
BP24	73	M	5m	1.18	4260	14.82	117.57	62	48	14	4	128
BP25	74	F	2m	1.19	1100	3.93	13.80	63	43	20	2	128
BP26	81	M	5y	1.46	2140	7.67	199.83	45	69	14	1	129
BP27	79	M	20d	0.74	5170	17.28	276.80	60	54	21	4	139
BP28	66	F	1m	0.16	2120	7.47	268.81	131	0	18	7	156
BP29	73	M	4m	1.86	4950	17.01	139.33	81	49	11	0	141
BP30	77	F	3m	1.58	2170	7.07	113.18	61	58	12	14	145

BP31	72	M	1m	0.43	2450	8.47	105.07	98	43	4	2	147
BP32	84	M	1m	6.87	6390	17.64	247.47	57	87	11	0	155
BP33	69	M	25y	0.59	3260	11.08	43.86	79	83	17	7	186
BP34	72	M	15d	1.56	6130	16.32	88.24	40	32	2	2	76
BP35	77	F	2m	1.75	1930	6.14	126.90	59	31	10	0	100
BP36	82	F	3m	6.97	6050	16.32	182.67	57	52	12	2	123
BP37	68	M	1m	5.94	1680	5.79	158.61	65	55	11	0	131
BP38	73	M	20d	1.72	4120	13.02	206.35	80	35	3	2	120
BP39	71	F	20d	7.41	14310	29.68	218.28	36	55	7	0	98
BP40	61	M	4m	0.59	2500	5.94	72.51	24	25	5	2	56
BP41	59	F	2m	0.58	9070	18.23	50.96	37	19	9	5	70
BP42	57	M	20d	1.67	1370	3.3	103.40	35	27	8	2	72
BP43	63	M	6m	0.12	1300	2.74	117.86	31	21	10	1	63
BP44	62	M	1y	0.66	1250	3.11	123.06	32	49	10	1	92
BP45	69	F	1y	0.05	2500	6.08	150	29	26	10	2	67
BP46	87	F	3m	0.07	6340	12.11	179.48	79	0	11	4	94
BP47	78	M	1m	1.24	4280	9.37	116.97	43	26	6	0	75
BP48	72	F	5m	0	1980	4.25	74.83	26	25	5	6	62

Figure S1

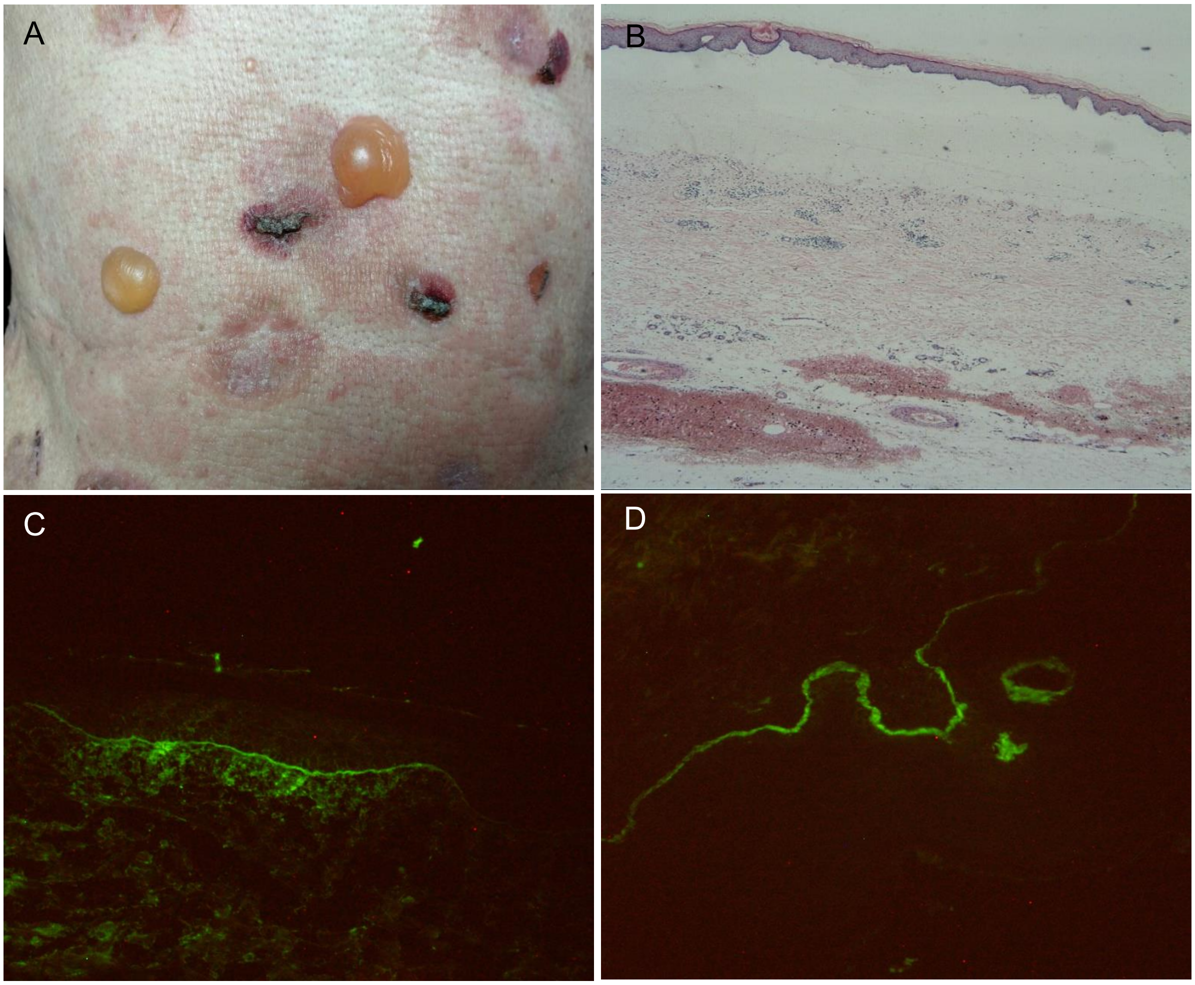

Figure S2

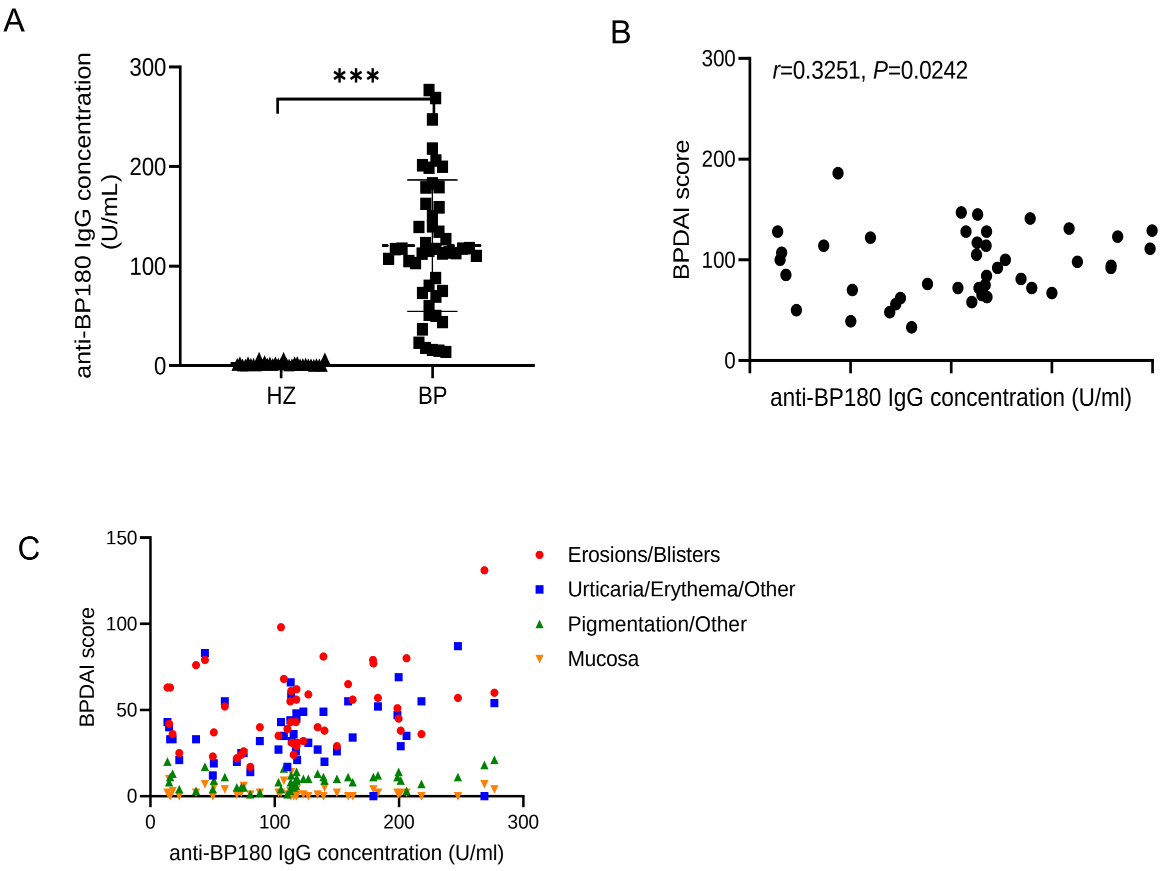

BPDAI parameters	Erosions/ blisters	Urticaria/ Erythema/ Other	Pigmentation/other	Mucosa
Pearson r	0.3402	0.1261	0.2797	-0.08332
P	0.0180	0.3931	0.0542	0.5734

Figure S3

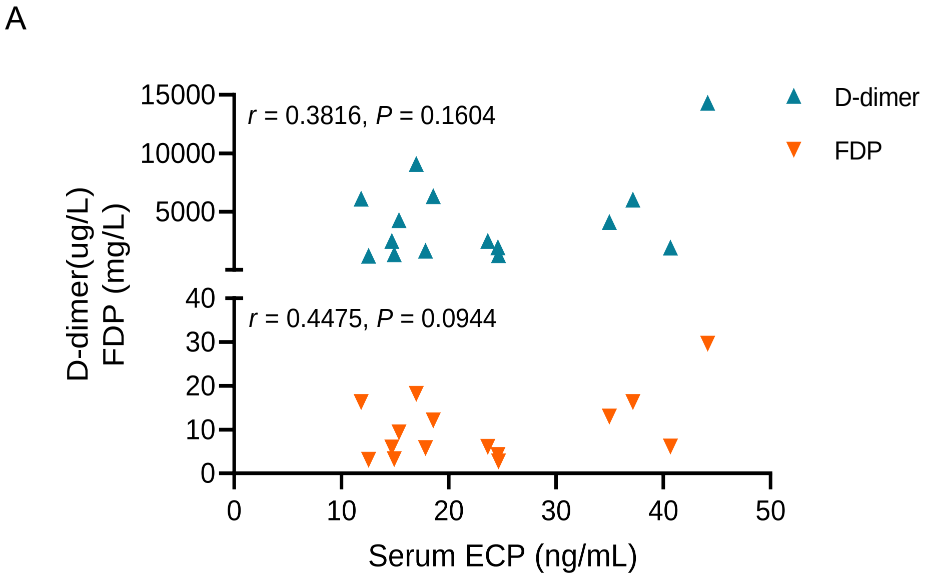

A
15000
10000
5000
40
30
20
10
0
r = 0.3816, P = 0.1604
r = 0.4475, P = 0.0944
D-dimer
FDP
D-dimer(ug/L)
FDP (mg/L)
0
10
20
30
40
50
Serum ECP (ng/mL)

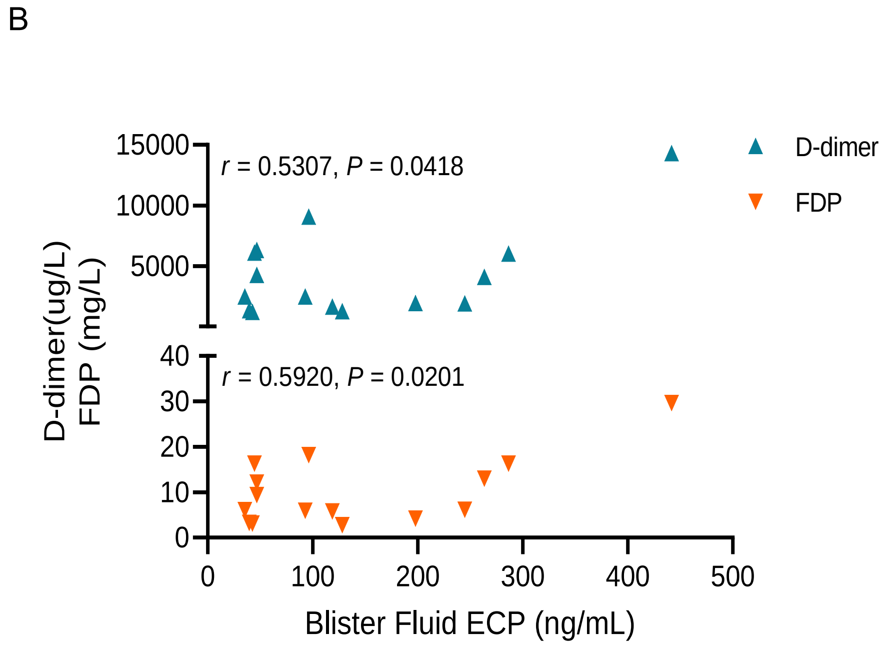

B
15000
10000
5000
40
30
20
10
0
r = 0.5307, P = 0.0418
r = 0.5920, P = 0.0201
D-dimer
FDP
D-dimer(ug/L)
FDP (mg/L)
0
100
200
300
400
500
Blister Fluid ECP (ng/mL)
